# Novel Approach to Dental Biofilm Management through Guided Biofilm Therapy (GBT): A Review

**DOI:** 10.3390/microorganisms9091966

**Published:** 2021-09-16

**Authors:** Deepti Shrivastava, Valentino Natoli, Kumar Chandan Srivastava, Ibrahim A Alzoubi, Ahmed Ismail Nagy, May Othman Hamza, Khalid Al-Johani, Mohammad Khursheed Alam, Zohaib Khurshid

**Affiliations:** 1 Periodontics, Department of Preventive Dentistry, College of Dentistry, Jouf University, Sakaka 72345, Saudi Arabia; dr.ibrahim.alzoubi@jodent.org; 2 Department of Dentistry, School of Biomedical and Health Sciences, European University of Madrid, 28670 Madrid, Spain; valentinonatoliodn@gmail.com; 3 Private Dental Practice, 72015 Fasano, Italy; 4 Oral Medicine Radiology, Department of Oral Maxillofacial Surgery Diagnostic Sciences, College of Dentistry, Jouf University, Sakaka 72345, Saudi Arabia; drkcs.omr@gmail.com; 5 Oral Surgery, Department of Oral Maxillofacial Surgery Diagnostic Sciences, Jouf University, Sakaka 72345, Saudi Arabia; dr.ahmed.nagy@jodent.org; 6 Department of Prosthodontics, College of Dentistry, Jouf University, Sakaka 72345, Saudi Arabia; dr.may.hamza@jodent.org; 7 Department of Oral Diagnostic Sciences, Faculty of Dentistry, King Abdulaziz University, Jeddah 21589, Saudi Arabia; Koalgehani@kau.edu.sa; 8 Orthodontics, Department of Preventive Dentistry, College of Dentistry, Jouf University, Sakaka 72345, Saudi Arabia; dralam@gmail.com; 9 Department of Prosthodontics and Dental Implantology, College of Dentistry, King Faisal University, Al-Ahsa 31982, Saudi Arabia; zsultan@kfu.edu.sa

**Keywords:** biofilm, air abrasives, disclosing agents, dental plaque, periodontitis, peri-implantitis

## Abstract

Dental biofilm plays a very crucial role in the etiopathogenesis of periodontal andperi-implant diseases. Over the past decade, tremendous research has been carried outto know the structure of biofilm and the mechanism by which it causes the destruction of supporting tissues of tooth or implant. Periodontal or peri-implant therapy usually begins with primarily removing thebiofilm and is considered as non-surgical mechanical debridement. Although scaling and root planing (SRP) is regarded as a gold standard for mechanical plaque debridement, various other means of biofilm removal have constantly been evolving. These may vary from different scaling systems such as vector systems to decontamination of pockets with LASER therapy. Nowadays, a new concept has emerged known as “guided biofilm therapy” (GBT). It is beneficial in removing the biofilm around the tooth and implant structures, resulting in better or comparable clinical outcomes than SRP. These results were substantiated with the reduction in the microbial load as well as the reduction in the inflammatory cytokines. This review will highlight the various aspects of GBT used in periodontal and peri-implant disease.

## 1. Introduction

Dental biofilm is a polymicrobial entity that resides on biotic and abiotic surfaces of the oral cavity [1]. These surfaces can range from hard or soft tissues of the oral cavity as well as the inanimate surfaces such as orthodontic bands, clear aligners, or prosthesis [2,3]. The supra and subgingival dental plaque biofilms can form on the tooth or implant surface. Being close to the gingival epithelium can deteriorate the periodontal and peri-implant health [3]. Dental plaque biofilms are also formed in some inaccessible regions of the oral cavity from where it is difficult to remove, thus compromising the home-care oral hygiene management. Although, scaling and root planing (SRP) is considered the gold standard for mechanical plaque debridement [4], it also has its own disadvantages [5,6,7]. Nowadays, an alternative novel approach is being practiced for removing the biofilm by visualizing it with a disclosing agent and subsequently removing it with specialized air abrasive powder. Lastly, it is followed by the removal of supra and subgingival calculus using specialized instruments. This concept has been named guided biofilm therapy (GBT) [8]. This review will explore the various aspects of GBT along with its substantial usage in the treatment of periodontal and peri-implant diseases.

## 2. Dental Biofilm and Its Relation to Periodontal and Peri-Implant Diseases

The oral cavity is an inhabitant of many microbial species, ranging from healthy microorganisms to those with pathogenic potential. The association between dental plaque and periodontal diseases is a well-established fact. However, until 1980, it was believed that the microorganism present in dental plaque remains in suspended or planktonic states [9]. Accordingly, the majority of the treatment was directed towards the removal of the dental plaque. Later, Casterton and colleagues have shown in their research that the microorganisms are not free-floating entities; rather, they are attached to the tooth surfaces [9]. Currently, it is well accepted that the microorganism lives in a complex environment known as biofilm [9,10]. It is known to contribute as the etiological agent for dental caries and periodontal disease [10,11]. A mature biofilm is a polymicrobial entity that primarily consists of bacteria, but it can also harbor protozoa, viruses, and fungi [12]. In 2002, Donlan and Casterton defined biofilm as a sessile microbiological community characterized by cells adhered to a substrate, to an interface, or each other, embedded in an extracellular polymeric substance matrix that produces and presents an altered phenotype, in terms of growth rate and gene transcription [13]. The biofilm has been considered as a single unit consisting of spatial arrangement of microorganism wherein the microorganisms display characteristics as a whole unit rather than a single entity [14]. Usually, the bacteria residing in the biofilm are considered beneficial bacteria and are known as commensal. However, during diminished host response predisposed by certain clinical situations, there is a shift in the composition of microbial flora where pathogenic bacterial species dominate over the healthy microbial flora. This phenomenon is known as “dysbiosis” [3]. The bacteria residing in the biofilm are responsible for the inflammatory cascade and, subsequently, the destruction of the supporting tissues [15,16]. Presently, periodontal and peri-implant disease are based on “polymicrobial synergy and dysbiosis” [17]. Thisis based on the hypothesis that the keystone pathogens such as *P. gingivalis* are initially introduced into the biofilm. Later, by undermining the host’s immunity, they succeed in modifying the composition of the microbial community, thus making it more pathogenic and competent to instigate disease [18]. These microbial alterations are accentuated by local environmental changes, thus establishing a microbiota capable of sustaining the dysbiosis and progressing the disease. It is also suggested that instead of directly causing the disease, the keystone pathogens bring about change in the metabolic activity of commensal which in turn increases the pathogenicity of the bacteria and thus manifests the disease [19]. The dysbiosis leads to an upsurge in the generation of inflammatory mediators, which triggers the host cell to produce toxic products. When these toxic products are produced, more than the threshold level leads to destruction of the tissues around the tooth and implant [3].

Additionally, the pathogenic bacteria trigger the innate immune response, which tries to cleanse the invading microorganism [20]. In the innate immune system, the pathogens trigger the Pattern Recognition Receptors (PRRs) that attach to the pathogen associated Molecular Patterns (PAMPs). These receptor types include toll-like receptors, nucleotide-binding oligomerization domain (NOD) proteins, cluster of differentiation 14 (CD14), complement receptor-3, lectins, and scavenger receptors [20,21]. The toll like receptors plays a crucial role in the progression of periodontal/peri-implant inflammation and bone resorption [22]. It has been reported that PAMPs activates T and B cells’ immune response leading to activation of cytokines and osteolytic pathway [22]. In conjunction with innate immunity, periodontal and peri-implant tissue produces various cytokines and chemokines which maintain the equilibrium.

However, in the presence of dysbiosis, there are certain cytokines such as IL-1β, tumor necrosis factor (TNF)-α and IL-6 which lead to destruction of the tissues [3]. Apart from these mechanisms, there are three protein pathways, namely nuclear factor kappa B (NF-κB), cyclo-oxygenase (COX) and lipo-oxygenase (LOX), which has an established role in the progression of periodontal/peri-implant diseases [3]. Hence, understanding its structure and biology is fundamental to unfold the etiopathogenesis behind the periodontal disease and peri-implant disease. Furthermore, researchers have found that 65% of infectious diseases are linked with the biofilm mode of growth of the microorganism [23].

The biofilm formed on the natural tooth or dental implant shares the common pattern of microbial colonization [24]. Within 30 min of implant insertion in the oral cavity, it is coated with a salivary pellicle and later becomescolonized with primary colonizers and subsequently with the late colonizers. Among the late colonizers, *Porphyromonasgingivalis (P. gingivalis*) and *Porphyromonasintermedia (*P.* intermedia)* are primarily responsible for peri-implantitis [25]. Surface roughness is a common feature incorporated in the implant to achieve osseointegration, but it also invites more biofilm microbial entities for colonization [24]. Biofilm formation is an inevitable phenomenon, but at the same time, its control and elimination cannot be overlooked, as it is the main culprit in the etiopathogenesis of periodontal or peri-implant diseases.

## 3. Rationale and Approaches for Non-Surgical Management of Dental Biofilm

The dental biofilm resides in the close vicinity ofthe gingiva epithelium. If proper oral hygiene measures are not taken care of, this supragingival biofilm will accumulate along the gingival epithelium and become a potential source ofgingival inflammation [3,15]. It is generally considered that the dental biofilm is noxious in nature, and if not disrupted it can progress to periodontitis, provided there is a simultaneous diminished host response [26,27]. In order to maintain periodontal stability after non-surgical periodontal therapy (NSPT) or surgical periodontal therapy, supportive periodontal therapy (SPT) plays an important role [28]. It is commonly observed that periodontal pockets can be easily recolonized with bacteria. Hence, regular recall visits in the form of periodontal maintenance therapy are of utmost importance [29]. Similarly, the biofilm formed on the dental implants has a similar microbiota as the neighboring tooth [30]. It is observed that the subgingival microbiota shares common periodontal pathogens as in periodontal disease. Hence, maintenance of the implant by removing the biofilm should be the principal management to combat the development of peri-mucositis or peri-implantitis.

Initially, based on the non-specific plaque hypothesis, the removal of the dental plaque was aimed at removing the bulk of the bacteria [31]. Later, the focus was shifted to specific bacterial removal based on specific plaque hypotheses [32]. Nevertheless, many hypotheses have been presented, but dental plaque remains a common etiological factor. Thus, a dental plaque was taken into consideration for the prevention of periodontal or peri-implant disease. Oral hygiene is maintained at home by personal care, which includes the usage of the toothbrush with dentifrices [26,33]. Despite meticulous cleaning, some amount of dental biofilm is left behind in undetected areas. Dental anatomical structures such as furcation, cervical enamel projection, deep groves, and concavities can be a potential ecological niche for bacteria [34]. Professional management of dental biofilm will enable professionals to reach inaccessible areas where dental plaque remains hidden. SRP is a gold standard in non-surgical mechanical debridement, based on the biofilm’s mechanical disruption [3]. Although it is a conventional treatment option, it has its own disadvantages such as being a time-consuming procedure, technically demanding, and occasionally uncomfortable to the patients [35]. After SRP, it has been reported that the lingual tooth surface and furcation areas are prone to residual calculus [6,7].

Moreover, furcation areas are seen to have incomplete root planing [7,36]. Additionally, gingival recession, and irreversible root damage has been reported if SRP is performed repeatedly as a protocol for supportive periodontal therapy(SPT) [5]. These untoward events will lead to dentinal hypersensitivity [37]. Furthermore, it has been observed that the outcome of SRP also depend on the skills of the operator [38]. Considering these drawbacks, various technologies and machines have been introduced to remove the dental biofilm, such as vector scaling systems, lasers, and an air polishing agent.

## 4. Guided Biofilm Therapy

**Figure 1:**
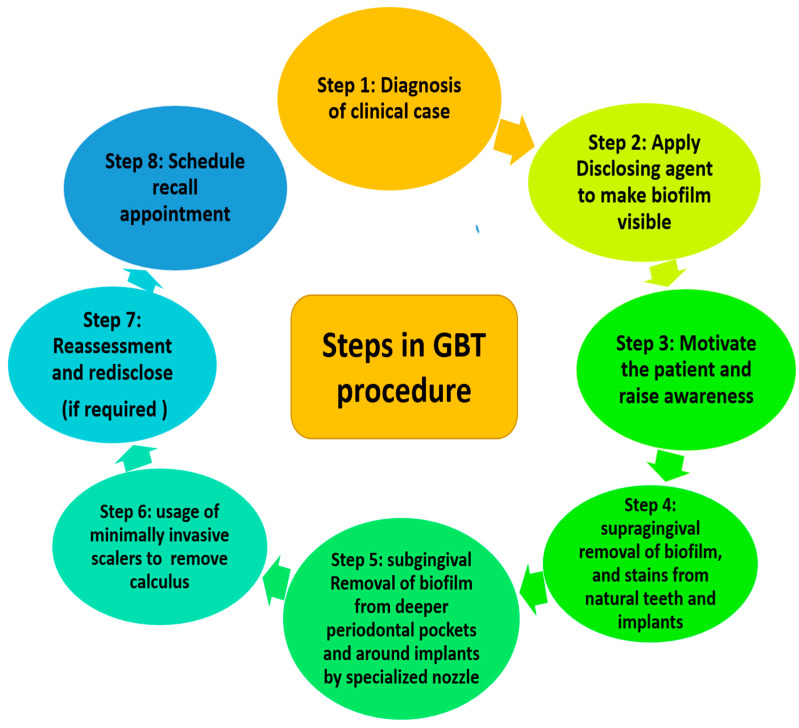
Steps in guided biofilm therapy (GBT).

**Figure 2:**
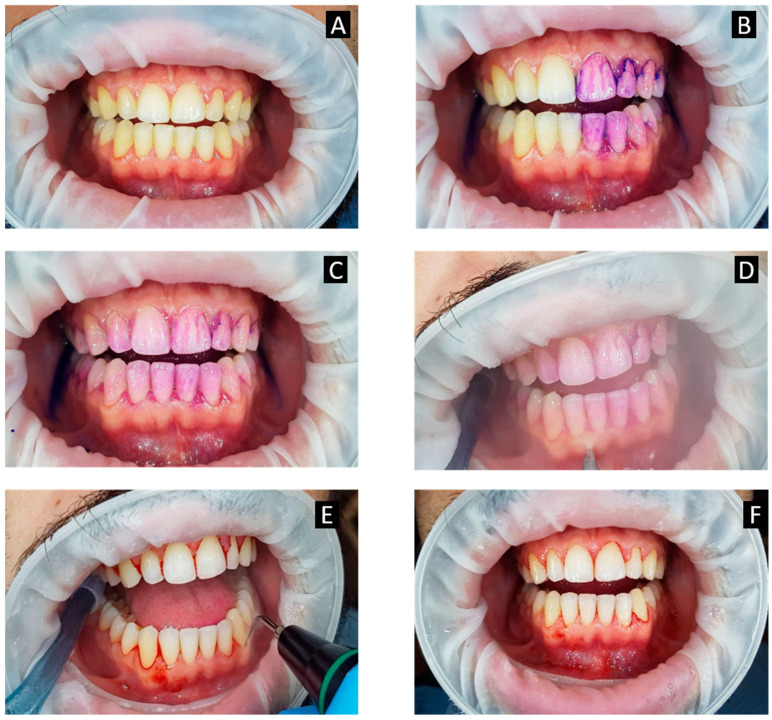
(**A**–**F**): Guided biofilm treatment (GBT) procedure in a patient with generalized bleeding on probing with plaque accumulation and localized calculus. (**A**) Pre-operative view; (**B**) application of disclosing agent on left half of the patient’s mouth; (**C**) application of disclosing agent over the patient’s entire mouth; (**D**) removal of supragingival biofilm and stains with air-polishing device (AIRFLOW^®^ Handy); (**E**) removal of the calculus removal from natural teeth with PIEZON^®^ PS allows thorough cleaning; (**F**) immediate post-operative view of the patient’s mouth after implementing the GBT protocol.

Guided biofilm therapy (GBT) is a new regimen where there is a sequential removal of plaque and calculus by initially detecting it with a disclosing agent followed by the usage of air abrasive powder for the removal of plaque and stains. Finally, the subgingival plaque and calculus are removed with a specialized nozzle and (if required) eventually scaling with a specialized tip is performed. The sequential steps of GBT are described in Figure 1. Similarly, the procedure performed on the patient has been elaborated in Figure 2.

## 5. Role of Disclosing Agent

The plaque detector or disclosing agent is a non-toxic substance that binds and colors bacterial plaque deposits to be visible and can be removed accurately. According to Wilkins (1959), a disclosing agent is a selective dye in solution and/or tablets used to visualize and identify dental biofilm on tooth surfaces [39]. The plaque detector helps the patient understand the state of dental hygiene at home and helps the clinician. It can be used during the active phase as well as in the professional hygiene maintenance phase. It will enable the clinician to follow the progress over time as well as provide an absolute certainty that all plaque deposits have been completely removed after periodontal therapy [40]. By applying the disclosing agents immediately before tooth brushing, SRP, or periodontal surgery, it is possible to precisely identify plaque and achieve a better degree of biofilm removal in terms of professional oral hygiene, thereby improving clinical results [40,41]. Many clinicians adopt disclosing agents to motivate and make patients’ oral hygiene home care performance measurable. This method has been given the name “colorimetric technique” [42]. The disclosing agent in the correct form and technique helps the patient perform better oral hygiene [43]. With the use of disclosing agents, the patients can clearly visualize the areas around the teeth, including the bacterial biofilm, thus facilitating the clinician to motivate patients to prevent periodontal disease.

In 1914, Skinner introduced the first disclosing agent (Skinner’s iodine solution) with the aim of teaching a home method of plaque identification [44]. At that time, it was not an established fact that the biofilm is the etiological agent for periodontal diseases and dental caries. Later, many monotonic dyes were introduced in the market, such as mercurochrome, Bismark brown, Genetian violet, and erythrosine [44]. Currently, various monotonic, bitonic and tritonic disclosing agents are available in the market. As the name suggests, the monotonic dyes highlight all deposits on dental surfaces with a single shade, whereas the bitonic compounds are able to differentiate between young and mature plaque. Recently, a new generation tritonal plaque detector gel has been introduced in the market. It is capable of identifying not only “young” and “mature” plaque, but also being the most acidic, it is capable of demineralizing the hard tissues of the tooth [45]. These are dispensed in various forms, including soluble tablets (mainly for home use) gel, solution, wafer, lozenges, mouth rinses, or pre-loaded pallets. On administration, these agents color the areas of the oral cavity where the biofilm is present, whereas the intensity of the color depends on the thickness of the plaque [46]. Ideally, a disclosing agent should be stable, odorless, tasteless, non-allergic, diffusible, and should adequately stain the tissue [47].

Likewise, in GBT the basic principle is to visualize the dental biofilm—the main etiologic agent for periodontal disease and peri-implant disease—and subsequently its removal with specialized instruments and equipment. These disclosing agents act as a professional guide to visualize the most inaccessible area of biofilm and thereby achieve mechanical plaque control by a minimally invasive procedure concept [8]. Many patients are visual learners and are impressed to view the plaque disclosed areas with the help of intraoral camera images on the computer monitor. This helps to motivate patients about the treatment and oral hygiene. The Electromedical system (EMS) provides the disclosing solution in pre-loaded pellets, in which the sponge is pre-soaked with disclosing agent. Hence, it can be easily applied on tooth surfaces with less effort and cleanliness.

There are some technical considerations to be kept in mind before applying the disclosing agents.

(a) It should be used with caution on the restorative material as it can cause staining;
(b) It should not be applied before the application of a sealant;
(c) Solutions containing alcohol should not be stored for more than 2 to 3 months as the alcohol will evaporate, making the solution too concentrated;
(d) Clinical assessments of soft tissue color, such as gingival status and gingival bleeding index, should be performed prior to the use of the plaque detector as dyeing the solution may mask the clinical status of the tissues;
(e) Always assess for any kind of allergies of patients before using the detectors in any form [44].

## 6. Air Polishing Devices

The usage of air polishing devices was introduced in 1945, wherein aluminum hydroxide [Al(OH)₃] powder was used for cavity preparation [48]. Various technical advances have improvised the devices, and currently, these air polishing devices are used for biofilm removal. The principle of air-polishing device was to deliver the slurry consisting of abrasive air particles (powder) mixed with water under pressure through a specialized nozzle [48].

The air-polishing device is based on two principles. First, the “venturi” powder chamber principle [49], where the powder mainly exits from the bottom of the chamber. In this technique the mixture of air and powder is created by the combination of carburetor technique and swirling [49]. In this technique, the amount of air powder discharged through the tube is dependent on the positioning of a sloping deflector at the filler cap [48]. As per the second principle, the air-powder slurry is formed by forcing the pressurized air into the powder chamber and reaching the outlet by swirling action [50]. The amount of powder emission is dependent on the screw setting. It has been observed that the amount of powder emission can be regulated by the first principle, whereas according to the second principle, the setting powder emission is inconsistent. In the second type, a decrease in the powder mass can decrease the powder output [48].

Currently, two systems(devices) are available for an air-polishing device: a hand-held device and a standalone device. These units are connected with the air turbine coupling of the dental unit. In the hand-held device, the powder chamber is smaller, thus requiring frequent refilling. Furthermore, the coupling unit of the hand-held device is bulkier. Hence, it is not ergonomic to use in inaccessible areas [48]. 

Essentially there are two types of nozzles used for air polishing, namely the supragingival and subgingival nozzle (Table 1). The supragingival nozzle, otherwise known as the standard nozzle, is basically used to remove the supragingival plaque and stains. On the other hand, subgingival nozzles can be used for treatment of periodontal pockets as well as in peri-implantitis. The supragingival nozzle is available at a 120 or 90° angle for posterior and anterior teeth, respectively [51]. Conversely, the subgingival nozzle is designed to have markings with either two outlets (Acteon) or three outlets (EMS). In the EMS system (PERIOFLOW^®^), the two outlets are located approximately 2 mm above the third nozzle which is situated at the tip. The outlets on the sides allow the exit of the air and powder mixture whereas the third outlet at the tip helps in the emergence of water [28]. It has been reported that a minute change in the size, diameter, length of the tube, and curvature can significantly affect the efficacy of the equipment [52]. It is also of prime importance to keep the nozzle at thecorrect distance from the tooth structure and the angulation of the slurry with the tooth surface. The incorrect angulation of the handpiece and distance from the tooth structure can have an adverse effect on soft tissues [53].

## 7. Air Abrasive Powders

The mechanism by which the air abrasive removes the biofilm, calculus, or tooth substance depends largely upon the particle size, mass hardness, and angularity of the abrasive delivered through the pressurized jet of water [48]. However, the increased pressure and water setting increase the efficacy of the instrument. Furthermore, it is also believed that water enhances the activity of air abrasive powder by removing the embedded particles on the surface. On the contrary, it is also believed that water film on the object will decrease the effect of air abrasives. Additionally, water’s kinetic energy will help break the particle and reduce its size, hence adversely affecting its efficiency [48].

Since the 1970s, various air abrasives have been used in clinical practice. Currently, sodium bicarbonate (NaHCO₃), glycine powder, erythritol powder, and bioactive glasses are some of the commercially available air abrasive powders [5]. These air abrasive powders mainly differ in particle size, shape, and consequently in their outcome. The particle size ranges from 1–250 µm with the glycine powder having a particle size of 45–60 µm and erythritol powder with a particle size of about 14–31 µm. The smallest particle size is found to be of bioactive glass (1–10 µm). Comparing the particle shape of air abrasives, NaHCO₃ has chiseled and sharp edges. The particle shape of glycine is similar to NaHCO₃, but it is less chiseled. Erythritol has extra fine grains whereas bioactive glass have has regular shape [5]. 

Another factor that can influence the treatment outcome is time duration. Instrumentation time is basically userdependent, and it may adversely affect the hard tissue or soft tissue if proper technique is not followed [48]. Additionally, the effectiveness of the technique is also modulated by the amount of powder present in the pressurized chamber. It has been reported that the usage of the slurry in the pressurized chamber decreases the efficacy and efficiency of the device [48].

### 7.1. Sodium Bicarbonate (NaHCO₃) 

It is a non-toxic, water-soluble powder used mainly for supragingival biofilm removal [5]. It has been reported that NaHCO₃ can alter the outer enamel layer, root cementum, and dentine [5,35] even if used for a shorter span of time. Furthermore, it is corrosive to certain restorative materials such as gold, amalgam, and composites [35]. Considering its abrasive nature, nowadays, low abrasive agents such as glycine and erythritols are commonly used [5]. However, in vitro studies pertaining to usage of NaHCO_3_ on Titanium disc has shown promising results as an air abrasive [55,56].

### 7.2. Glycine Powder

Glycine is an amino acid, consisting of non-toxic, biocompatible organic salt crystals which have slow solubility in water. It is approximately 80% less abrasive than NaHCO₃, and accordingly, studies have reported less soft tissue damage using glycine powder. Rarely, air emphysemas have been reported as an adverse reaction which wasresolved within four days [57].

### 7.3. Erythitol Powder

It is an artificial sweetener and a food additive. It is chemically neutral, non-toxic, water-soluble polyol. Compared to glycine, it has a smaller particle size and is more stable [58]. Its usage in periodontitis patients has been reported to lower the counts of *P. gingivalis* [58] and is more acceptable and tolerant to the patients [59,60]. Some authors have reported that erythritol powder causedno significant damage to soft or hard tissue after using erythritol powder. Furthermore, erythritol powder showed a smooth surface on dentin compared with NaHCO₃ and glycine powder. It was also found in a 12 month follow-up period that erythritol powder resulted in significant reduction in probing pocket depth (PPD) and bleeding on probing (BOP) [61].

## 8. Guided Biofilm Therapy in Periodontal Disease and Peri-Implant Disease

The literature search shows many studies have been performed to assess the outcome of GBT on periodontal or peri-implant disease. Few studies have reported reduction in the red-complex bacteria when periodontally healthy individuals underwent GBT treatment [62]. In addition, studies with periodontitis patients have also found significant reduction in the pocket depth of more than 5 mm along with the reduction in *Tannerella* *forsythia* (*T. forsythia)* and *Treponemadenticola* (*T. denticola*) with subgingival usage of erythritol as air polishing powder along with reduction in Matrix metalloproteinases (MMP-8) [63]. Furthermore, in another study a significant decrease in the levels of *P. gingivalis* was reported after one month in the group treated with erythritol air-polishing compared to SRP [35]. Contrary to this, there are studies which have observed the same or lesser clinical outcomes of glycine/erythritol air-polishing compared to SRP [64,65,66,67].

Home care oral hygiene alone does nothave the ability to completely remove the newly formed bacterial deposits from the residual pockets, which is a well-established fact. Hence, patients are supposed to be put on SPT that needs professional dental biofilm management [61,68]. GBT has been proven to be as effective as conventional SRP treatment in clinical outcomes [61,65,66]. However, GBT was reported to be more comfortable to patients with less pain perception [61,69]. In another study, the 12 month post-operative count of *Aggregatibacter actinomycetemcomitas* (*A. actinomycetemcomitans)* was less at the test site treated with erythritol with 3% chlorhexidine than the control site receiving SRP. However, the role of adding chlorhexidine to erythritol cannot be substantiated with the reduction of bacteria.

Additionally, it has not caused any harm to the soft tissues [61]. A systematic review and meta-analysis found that air polishing devices are safe and effective in carrying out biofilm removal similar to conventional therapy when used in SPT [28]. It was concluded that the main advantage of air polishing in supportive periodontal therapy is that it does notcause harm to soft tissue, tooth structure, or root structure. Moreover, it has better compliance among patients and consumes less time. In a recently conducted retrospective study, the clinical outcomes with low abrasive air powder glycine were equally effective as conventional mechanical debridement during SPT. It was also suggested that it should be restricted in the area of furcation where SRP is advisable [69].

Dental implants have emerged as effective rehabilitation management for a non-restorable tooth or replacement of tooth in a missing area with a reported success rate of 97% in a follow-up period of 10 years [70]. However, biological complications such as peri-implant disease are reported with a prevalence of 46.83 and 19.83% for peri-implant mucositis and peri-implantitis, respectively [70]. A plaque score of ≥30% is a risk indicator for peri-implant mucositis, and similarly, a plaque score of ≥25% is associated with peri-implantitis [71,72].

As per the consensus report, conventional non-surgical mechanical therapy and oral hygiene reinforcement are the standard treatment for peri-implant mucositis. This treatment will help in the reduction of PPD of approximately 0.5–1.0 mm and 15–40% reduction in BOP. On the other hand, NSPT of peri-implantitis usually helps reduce BOP by 20–50% and, in some cases pocket reduction of ≤1 mm. However, in advanced cases complete resolution of disease is unlikely with mechanical plaque control [73]. Nonetheless, mechanical plaque control remains a mainstay in the treatment of peri-implant disease or during supportive therapy following implant insertion [74]. As per the consensus statement 2016, the air-polishing device has shown positive clinical outcome for peri-implant mucositis or peri-implantitis [75]. Following a non-surgical management of peri-implant mucositis or peri-implantitis, a significant reduction in BOP and bleeding index was found when glycine powder was used as monotherapy or adjunctive measure [75]. In an animal model study, partial regeneration and less inflammation were reported [76]. Additionally, studies have reported a statistically significant result with an air-polishing device either with glycine or erythritol in the treatment of peri-implant diseases [77,78,79,80]. Contrary to this, studies have reported either similar or no additional benefit over SRP [81,82,83,84,85]. Few fundamental studies related to usage of air polishing powder in periodontal and peri-implant disease have been summarized in Table 2.

The GBT concept may have the following advantages over the conventional methods of prophylaxis:

1. The use of a plaque disclosing agent allows the operator to determine the patient compliance in executing proper oral hygiene practices. It also allows the patient to visualize areas that were neglected;
2. The use of an air-polishing device can remove the disclosed plaque effectively and safely without causing soft tissue damage compared to conventional rubber cups, especially during subgingival plaque removal;
3. The removal of plaque using air polishing prior to ultrasonic scaling provides better visible access to calculus deposits. Instead of the indiscriminate use of ultrasonic scalers for the entire dentition, the operator can now target the use of ultrasonic scalers on sites with mineralized deposits. This minimizes soft tissue damage and CAL caused by ultrasonic scaling at sites with shallow pocket depths. From the patient’s perspective, this translates to lesser discomfort and sensitivity experienced during ultrasonic scaling. Overall, treatment time is also reduced;
4. A second plaque disclosure provides quality control and assurance to the patient as well as the operator.

## 9. Limitations and Future Recommendation

Guided biofilm therapy with glycine or erythritol powder has shown better acceptance in a patient with less pain perception in periodontal treatment. Since periodontal therapy needs constant monitoring, reassessment, and treatment which is given as supportive, periodontal therapy seems to be more acceptable to patients. With the application of a disclosing agent, it is much easier to visualize the plaque and hence helps in time management with more ergonomic benefits and less fatigue. However, the effectiveness of the procedure after three months is diminished as seen similar to SRP; thus, a long-term study should be conducted to assess the clinical outcome along with the biochemical and microbiological assessment. Furthermore, its effect on patients with systemic disease is lacking and hence it should be evaluated in long term studies.

Similarly, in peri-implant diseases, glycine powder has successfully helped in reduction of bleeding on probing and bleeding index. Pertaining to peri-implantitis, studies have shown comparable or lesser clinical outcome toSRP in the non-surgical management. Hence a long-term study with biochemical and microbiological assessment should be conducted to providean insight about the potential clinical outcome gain.

Few of the limitations mentioned in the consensus reports of periodontal and peri-implant disease werewith the usage of glycine powder. Hence a systematic review with erythritol air abrasive powder should be conducted to analyze the effect of erythritol powder. Additionally, studies should be conducted, keeping in mind about the sequential steps of GBT.

## 10. Conclusions

With the current evidence, it can be concluded that GBT is an effective means of removing biofilm from the tooth or implant vicinity. Compared to SRP, GBT was reported with better patient compliance and less pain perception in non-surgical periodontal therapy or supportive periodontal therapy. Although, in peri-implant diseases, it does help in the reduction of plaque, its usage as monotherapy needs further investigation with long term studies as the clinical outcome is short-lasting.

## Figures and Tables

**Table 1:** Technical tips of air-polishing device with air abrasive powder [54].

FEATURES	STANDARD NOZZLE	SUBGINGIVAL NOZZLE
USE	Supragingival and shallow subgingival (≥4 mm)	The United States Food and Drug Administration has approved these devices for subgingival use in periodontal pockets up to 5 mm in the U.S., and Health Canada has approved them for up to 10 mm in Canada.
TECHNIQUE	Position 3 mm away from the tooth and angled between 30–60° to labial surface of the anterior teeth. For the posterior teeth, it should be kept 80° for buccal surface and 90° for the occlusal surface.	Insert nozzle tip to the bottom of the pocket and pull the nozzle back 1 mm and later activate the spray.
MOTION	Move in a continuous half-circle “smiley face” motion progressing of the tooth (3–5 s) or up and down vertical stroke.	Move nozzle continuously in a vertical-incisal motion to cover the entire length until removed from pocket for about 5 s.

**Table 2:** Studies with glycine/erythritol as an air-polishing agent in the treatment of periodontal diseases/peri-implant diseases.

S. No	Author, Year	Objective	Subjects	Sample Size	Parameters	Outcome
1	Park, E.J. et al., 2018 [34]	Comparison of erythritol powder air-polishing device (EPAP) as a supplement to SRP therapy.	Human	Split mouth study design with twenty-one patients of moderate chronic periodontitis.	All patients received SRP (control) SRP+EPAP (test) on either jaw. Clinical and microbiological parameters were examined before treatment, 1 and 3 months post treatment.	Clinical parameters showed no significant difference between groups. However, counts of *P. gingivalis* were significantly lower in the test group at 1 month follow up period. Both parameters deteriorated at 3 months.
2	Caygur, A. et al., 2017 [59]	Comparison of glycine powder air-polishing (GPAP)combined with SRP in the treatment of periodontitis and halitosis.	Human	Randomized clinical trial with sixty chronic periodontitis patients.	Patients were randomly allocated into control (SRP) and test group (SRP + GPAP). Clinical parameters were recorded at baseline and 1 month post treatment; also, the volatile sulphur compounds at baseline, immediately after treatment, and at 7, 14, and 30 days.	Clinical parameters were significantly reduced in both groups. The volatile sulphur compounds (VSCs) were significantly different at 1 month compared with baseline in both groups. GPAP has no additional benefit and is shown equally effective.
3	Hägi, T.T. et al., 2013 [60]	Comparison of erythritol powder by means of an air-polishing (EPAP) device and of (SRP) during SPT up to 3 months.	Human	Randomized clinical trial with forty patients on SPT, after completion of active treatment of moderate or severe periodontitis.	Patients were randomly assigned to control and test group. Clinical parameters such as plaque indices, BOP, PPD, and CAL were recorded at baseline and at 3 months. Patient’s comfort using a visual analog scale was also recorded.	All clinical parameters showed non-significant improvement. However, patients in test group showed significantly lower visual analogue scale (VAS) scores.
4	Müller, N. et al., 2014 [61]	Comparison of repeated subgingival air-polishing with a new erythritol powder containing 0.3% chlorhexidine with conventional ultrasonic debridement over 12 months.	Human	Randomized, parallel arm clinical trial with fifty patients on SPT.	Fifty patients were treated with subgingival air-polishing (test side) or ultrasonic debridement (control side) and were monitored at an interval of 3-month intervals up to 12 months.	Non-significant difference in clinical parameters was seen between the study groups. Test group showed significantly lesser count of *A.* *actinomycetemcomitas* at 12 months.
5	Reinhardt, B. et al., 2019 [62]	Comparison of periodontal pathogens of red complex after supragingival debridement (SD) with adjunctive full mouth (FM-GPAP) in periodontal healthy individuals.	Human	Randomized, split mouth study design with eighty-seven.	Subjects with 87 medically and periodontally healthy intraoral carriers of red complex bacteria were randomly assigned to receive SD with adjunctive FM-GPAP (test) or SD alone (control). Microbiological samples were obtained at baseline, and two, five, and nine days following intervention.	The count of red complex bacteria was significantly less in the test group in comparision to the control group following treatment and at day 9.However, the values were similar to baseline values when observed at 6 and 12 weeks.
6	Jentsch, H.F. et al., 2020 [63]	Comparison of adjunctive use of EPAPduring subgingival instrumentation (SI) with conventional NSPT.	Human	Randomized clinical trial with forty-two patients with moderate to severe periodontitis.	Patients were randomly assigned to control and test group receiving two different approaches of non-surgical periodontal therapy by SI, where test group additionally received EPAP. Clinical parameters, biomarkers and microorganism were measured at baseline, three and six months after SI.	Clinical parameters showed significant improvement at 2 and 6 months. However, test group showed more sites with PD ≥ 5 mm after six months. Significant reduction in the *T. forsythia counts* and *T. denticola* along with lesser values of matrix metalloprotienases -8 in the test group.
7	Hägi, T.T. et al., 2015 [64]	Clinical efficacy of low abrasive EPAP over a period of 6 months in patients undergoing SPT.	Human	Randomized clinical trial with forty chronic periodontitis patients.	Patients were randomly assigned to control (SRP) and test group (subgingival EPAP). Clinical parameters were evaluated at baseline, 3, and 6 month intervals. Site considered for evaluation had BOP with PPD of ≥ 4 mm	A significant reduction of BOP, PPD and increase of CAL was observed between groups at 3 month intervals, but no significant difference at 6 months. No major change in periodontal pathogens recorded.
8	Tsang, Y.C. et al., 2018 [65]	Evaluation of GPAP as NSPT in subjects with chronic periodontitis.	Human	Randomized, split mouth study design with twenty-seven chronic periodontitis patients.	Patients received SRP and GPAP (test group) or SRP and air flushing with water (control group) at sites with PPD of ≥5 mm. Clinical parameters, gingival crevicular fluid(GCF) volumes, and the concentrations of interleukin-1β (IL-1β)and interleukin-1ra(IL-1ra) in GCF were measured at baseline and one, three, and six months after the intervention.	Significant improvements were recorded in clinical parameters in both groups. No significant difference in GCF levels of IL-1β and IL-1ra) were seen between the groups.
9	Kargas, K. et al., 2015 [66]	To evaluate the efficiency of subgingival GPAP during SPT.	Human	Randomized, split mouth study design with twenty-five chronic periodontitis patients.	Patients were randomly allocated to group receiving SRP with hand instruments, GPAP, subgingival ultrasonic debridement (UD), and no subgingival treatment (NT). Clinical parameters were recorded at baseline, three, and sixmonths. Subgingival samples were taken for microbiological analysis.	Clinically and microbiologicaly GPAP has no additional benefits over SRP or subgingival ultrasonic scaling.
10	Flemmig, T.F. et al., 2012 [67]	Comparison of supragingivally (GPAP) with conventional SRP in patients with in moderate-to-deep periodontal pockets.	Human	Randomized clinical trial with thirty patients with chronic periodontitis.	Patients were randomly allocated to received (FM- GPAP) or(SRP) followed by coronal polishing Patients rinsed with 0.12% chlorhexidine gluconate after debridement, and twice daily, for 2 weeks.	Test group showed significantly lesser total viable bacterial counts in chronic periodontitis patients when compared to SRP immediately after debridement and at the tenth day.
11	Wennström, J.L. et al., 2011 [68]	Comparison of subgingival air polishing (AP) compared with UD during SPT.	Human	Randomized, split mouth study design with twenty patients on SPT	Patients were randomly assigned two different subgingival debridement treatment groups—GPAP specially designed nozzle (test) and ultrasonic instrumentation (control). Clinical parameters and microbiological were recorded at baseline, fourteen, and sixtydays.	Results: both treatment procedures resulted in significant reductions in clinical parameters—BOP, PPD and relative attachment level at 2 months. Perceived treatment discomfort was less for AP than UD.
12	Solderer, A. et al. 2020 [76]	Comparison of mechanical debridement with/without air polishing on the healing of induced peri-implantitis.	Dogs	Non-randomized, animal study with forty-eight mandibular implants.	Depending on the study group, specific surgical cleaning approach is adopted along with augmentation procedure. Debridement followed by guided bone regeneration (GBR) Air polishing cleaning using an experimental sterile powder followed by GBR Only debridement followed by air polishing. Combination of all above approaches Histological measurements of the relative bone gain; depth of the defect, remaining bone, and soft tissue was measured.	Non-significant partial regeneration was observed in all treatment approaches. However, pre-treatment with air polishing showed less inflammation.
13	Menini et al., 2019 [77]	Comparison of the cleaning efficacy of GPAP against two different professional oral hygiene techniques on implants supporting full-arch fixed prostheses.	Human	Randomized, split mouth study design with thirty patients with a total of 32 implant fixed full arch rehabilitations in the maxilla and/or mandible (134 implants).	Patients randomly assigned by following a splitmouth method: all the patients received glycine air polishing (G) in one side of the arch (*n* = 32), and sodium bicarbonate air polishing (B) (*n* = 16) or manual scaling with carbon-fiber curette (C) (*n* = 16) was performed in the opposite side. After the hygiene procedures, plaque index and spontaneous bleeding were recorded.	Plaque index reduction was significantly more for group treated with GPAP and sodium bicarbonate air polishing compared to manual scaling. Group treated with sodium bicarbonate were having maximum spontaneous bleeding as compared to other groups. It was concluded that the professional oral hygiene on implants using GPAP showed better patient acceptance and cleaning.
14	Siena et al., 2015 [78]	Comparative evaluation of professional oral hygiene with or without the adjunct of GPAP for the treatment of peri-implant mucositis	Human	Non-randomized clinical trial on 30 patients with peri implant mucositis	30 patients were allocated into two groups. first group received professional oral hygiene manoeuvres (POH) while in the test group, received the GPAP.PPD, bleeding index (BI) and plaque index (PI) were measured at baseline, three, and six months.	The present reports showed that both techniques were useful for the treatment of peri-implant mucositis. In the test group (with glycine powder), a significant reduction inprobing depth was observed.
15	Lupi, S.M. et al. 2017 [79]	The study evaluated the efficacy of maintenance treatment with glycine powder on the periodontal health of peri-implant tissues.	Human	Single-masked, randomized clinical intervention trial on 46 patients with partial or total edentulism with 88 implants.	46 patients with 88 implants were randomly assigned into two groups treated with either an air abrasive with the (GPAP) or to a manual debridement and chlorhexidine administration treatment group (MDA). Clinical data were collected at 0, 3, and 6 month intervals. PI, BOP, PPD, CAL, and bleeding score (BS) were analyzed.	Within the limits of the study, treatment with glycine seems appropriate in the maintenance of peri-implant health and more effective than the traditional treatment with plastic curette and chlorhexidine.
16	John, G. et al., 2015 [80]	Evaluation of the effectiveness of an air-abrasive device (AAD) for non-surgical treatment of peri-implantitis.	Human	Prospective, parallel grouped, randomized controlled clinical trial on twenty five patients with initial to moderate peri-implantitis.	25 patients, with initial to moderate peri-implantitis in one implant, underwent an oral hygiene program and were randomly treated using either AAD (amino acid glycine powder) or mechanical debridement using carbon curettes and MDA. Clinical parameters were measured at baseline and tweleve months.	The present study has indicated that both treatment procedures resulted in comparable but limited CAL gains at 12 months. Furthermore, it could be detected that AAD was associated with significantly higher BOP decrease than MDA. Thus, AAD seems to be better than MDA.
17	Ji, Y.J. et al., 2102 [81]	This pilot clinical trial evaluated the effect of GPAP as an adjunct in treating peri-implant mucositis.	Human	Randomized clinical trial with twenty-four patients with peri-implant mucositis.	Twenty-four peri-implant mucositis patients were randomly assigned to test (12 subjects with 17 implants) and control (12 subjects with 16 implants) groups. In the test group, the sites with PPD of 4 mm were additionally treated by GPAP for 5 sec. Clinical parameters were measured at 1-week, one-month, and three-month recall visits.	At the 3-month visit, there was no significant difference existing between two groups in probing depth. This pilot clinical trial suggests that NSPT may be beneficial for treatment of peri-implant mucositis. However, adjunctive GPAP treatment seems to have a minimal beneficial effect.
18	Al Ghazal, L. et al., 2017 [82]	Comparing the two different methods of debridement for improving peri-implant soft tissue health for a follow up period of 12 months.	Human	Randomized, single blinded, parallel group clinical trial with twenty patients (25 implants.	20 patients with no signs of pathologic bone loss around implants (25 implants) were selected. Patients were scheduled to be reviewed at 0, 3, 6, 9, and 12 months. Nine patients (15 implants) were randomly allocated to a test group (Air-FlowVR Perio, EMS) (AFP) and control group comprised of nine patients (10 implants) which were treated with titanium curettes (TC). Peri-implant GCF samples were analyzed to quantitatively measure the concentration of six interleukins.	The present study showed that both the treatment methods were effective in reducing perimplant inflammation with no difference in clinical parameter such as BOP. The present study showed a significant relationship between IL-6 and BOP.
19	Sahm, H. et al., 2011 [83]	To evaluate the effectiveness of an AAD for NST of peri-implantitis.	Human	Prospective, parallel group designed, randomized controlled clinical study with 30 patients of initial to moderate peri- implantitis.	Thirty patients, each of whom displayed at least one implant with initial to moderate peri-implantitis, were enrolled in an oral hygiene program (OHP) and randomly instrumented using either (1) AAD or (2) mechanical debridement using carbon curets and MDA. Clinical parameters were measured at baseline, 3, and 6 months after treatment [e.g., BOP, PPD, CAL].	The present study concluded that both treatment procedures resulted in comparable but limited CAL gains at 6 months, and OHP+AAD was associated with significantly higher BOP reductions than OHP+MDA.
20	Persson, G.R.,et al., 2011 [84]	Clinical and microbiological NST of peri-implantitis lesions using either an erbium-doped: yttrium, aluminum, and garnet (Er:YAG) laser or an air-abrasive subgingival polishing method.	Human	Non-randomized clinical trial with 42 patients with peri-implantitis.	42 patients with peri-implantitis were treated at one time with an Er:YAG laser or an air-abrasive device. Baseline and 6-month intraoral radiographs were assessed with a software program. The checkerboard DNA–DNA hybridization method was used to assess 74 bacterial species from the site with the deepest probing depth (PD) at the implant.	Non-significant probing depth reduction was seen in both the groups. No baseline differences in bacterial counts between groups were found. In the air-abrasive group, *Pseudomonas aeruginosa*, *Staphylococcus aureus*, and *Staphylococcus anaerobius* were found at lower counts at 1 month after therapy. Six-month data demonstrated that both methods failed to reduce bacterial counts.
21	Hentenaar, D.F. et al., 2021 [85]	Comparison of erythritol air polishing with piezoelectric ultrasonic scaling in the non-surgical treatment of peri-implantitis.	Human	Randomized clinical trial with eight patients of peri-implantitis having 139 implants.	80 patients (n = 139 implants) with peri-implantitis PPD ≥5 mm, marginal bone loss (MBL) ≥2 mm as compared to bone level at implant placement, bleeding, and/or suppuration on probing (BOP/SOP)) were randomly allocated to EPAP or ultrasonic treatment. Clinical outcome and pain/discomfort VAS were measure at 0, 3,6,9,12 months.	Three months after therapy, no significant difference in mean BOP, plaque score, PPD, MBL between the EPAP and ultrasonic group. Pain/discomfort was low in both groups. EPAP seems as effective as piezoelectric ultrasonic scaling in the NST of peri-implantitis.

## References

[1] Sanz M., Beighton D., Curtis M.A., Cury J.A., Dige I., Dommisch H., Ellwood R., Giacaman R.A., Herrera D., Herzberg M.C. (2017). Role of microbial biofilms in the maintenance of oral health and in the development of dental caries and perio-dontal diseases. Consensus report of group 1 of the Joint EFP/ORCA workshop on the boundaries between caries and peri-odontal disease. J. Clin. Periodontol..

[2] Meto A., Colombari B., Castagnoli A., Sarti M., Denti L., Blasi E. (2019). Efficacy of a Copper–Calcium–Hydroxide Solution in Reducing Microbial Plaque on Orthodontic Clear Aligners: A Case Report. Eur. J. Dent..

[3] Lasserre J.F., Brecx M.C., Toma S. (2018). Oral Microbes, Biofilms and Their Role in Periodontal and Peri-Implant Diseases. Materials.

[4] Lindhe J., Westfelt E., Nyman S., Socransky S.S., Haffajee A.D. (1984). Long-term effect of surgical/non-surgical treatment of periodontal disease. J. Clin. Periodontol..

[5] Sultan D.A., Hill R.G., Gillam D.G. (2017). Air-polishing in subgingival root debridement: A critical literature review. J. Dent. Oral Biol..

[6] Rabbani G.M., Ash M.M., Caffesse R.G. (1981). The effectiveness of subgingival scaling and root planing in calculus removal. J. Periodontol..

[7] Eaton K.A., Kieser J.B., Davies R.M. (1985). The removal of root surface deposits. J. Clin. Periodontol..

[8] Mensi M., Scotti E., Sordillo A., Agosti R., Calza S. (2020). Plaque disclosing agent as a guide for professional biofilm removal: A randomized controlled clinical trial. Int. J. Dent. Hyg..

[9] Seneviratne C.J., Zhang C.F., Samaranayake L.P. (2011). Dental plaque biofilm in oral health and disease. Chin. J. Dent. Res..

[10] Marsh P.D., Zaura E. (2017). Dental biofilm: Ecological interactions in health and disease. J. Clin. Periodontol..

[11] Nimbulkar G., Garacha V., Shetty V., Bhor K., Srivastava K.C., Shrivastava D., Sghaireen M.G. (2020). Microbiological and Clinical evaluation of Neem gel and Chlorhexidine gel on Dental Plaque and Gingivitis in 20–30 Years Old Adults: A Randomized Parallel-Armed, Double-Blinded Controlled Trial. J. Pharm. Bioallied. Sci..

[12] Larsen T., Fiehn N.E. (2017). Dental biofilm infections-an update. APMIS.

[13] Rode Sde M., Gimenez X., Montoya V.C., Gómez M., Blanc S.L., Medina M., Salinas E., Pedroza J., Zaldivar-Chiapa R.M., Pannuti C.M. (2012). Daily biofilm control and oral health: Consensus on the epidemiological challenge-Latin American Advisory Panel. Braz. Oral Res..

[14] Berger D., Rakhamimova A., Pollack A., Loewy Z. (2018). Oral Biofilms: Development, Control, and Analysis. High Throughput.

[15] Shrivastava D., Srivastava K.C., Ganji K.K., Alam M.K., Al Zoubi I., Sghaireen M.G. (2021). Quantitative Assessment of Gingival Inflammation in Patients Undergoing Nonsurgical Periodontal Therapy Using Photometric CIELab Analysis. BioMed Res. Int..

[16] Shrivastava D., Srivastava K.C., Dayakara J.K., Sghaireen M.G., Gudipaneni R.K., Al-Johani K., Baig M.N., Khurshid Z. (2020). BactericidalActivity of Crevicular Polymorphonuclear Neutrophils in Chronic Periodontitis Patients and Healthy Subjects under the Influence of Areca Nut Extract: An In Vitro Study. Appl. Sci..

[17] Hajishengallis G., Lamont R.J. (2012). Beyond the red complex and into more complexity: The polymicrobial synergy and dysbiosis (PSD) model of periodontal disease etiology. Mol. Oral Microbiol..

[18] Hajishengallis G., Darveau R.P., Curtis M.A. (2012). The keystone-pathogen hypothesis. Nat. Rev. Microbiol..

[19] Darveau R.P. (2010). Periodontitis: A polymicrobial disruption of host homeostasis. Nat. Rev. Microbiol..

[20] Silva N., Abusleme L., Bravo D., Dutzan N., Garcia-Sesnich J., Vernal R., Hernandez M., Gamonal J. (2015). Host response mechanisms in periodontal diseases. J. Appl. Oral Sci..

[21] Amano A. (2010). Host-parasite interactions in periodontitis: Microbial pathogenicity and innate immunity. Periodontology 2000.

[22] Kajiya M., Kurihara H. (2021). Molecular Mechanisms of Periodontal Disease. Int. J. Mol. Sci..

[23] Jamal M., Ahmad W., Andleeb S., Jalil F., Imran M., Nawaz M.A., Hussain T., Ali M., Rafiq M., Kamil M.A. (2018). Bacterial biofilm and associated infections. J. Chin. Med. Assoc..

[24] Dhir S. (2013). Biofilm and dental implant: The microbial link. J. Indian Soc. Periodontol..

[25] Mombelli A., Lang N.P. (1994). Microbial aspects of implant dentistry. Periodontology 2000.

[26] Sahni K., Khashai F., Forghany A., Krasieva T., Wilder-Smith P. (2016). Exploring Mechanisms of Biofilm Removal. Dentistry (Sunnyvale).

[27] Fatima T., Khurshid Z., Rehman A., Imran E., Srivastava K.C., Shrivastava D. (2021). Gingival Crevicular Fluid (GCF): A Diagnostic Tool for the Detection of Periodontal Health and Diseases. Molecules.

[28] Ng E., Byun R., Spahr A., Divnic-Resnik T. (2018). The efficacy of air polishing devices in supportive periodontal therapy: A systematic review and meta-analysis. Quintessence Int..

[29] Renvert S., Persson G.R. (2004). Supportive periodontal therapy. Periodontology 2000.

[30] Cortés-Acha B., Figueiredo R., Seminago R., Roig F.J., Llorens C., Valmaseda-Castellón E. (2017). Microbiota Analysis of Biofilms on Experimental Abutments Mimicking Dental Implants: An In Vivo Model. J. Periodontol..

[31] Schultz-Haudt S., Bruce M.A., Bibby B.G. (1954). Bacterial factors in nonspecific gingivitis. J. Dent. Res..

[32] Loesche W.J. (1979). Clinical and microbiological aspects of chemotherapeutic agents used according to the specific plaque hypothesis. J. Dent. Res..

[33] Meto A., Colombari B., Odorici A., Giva L.B., Pericolini E., Regina A.L., Blasi E. (2020). Antibacterial Effects of MicroRepair® BIOMA-Based Toothpaste and Chewing Gum on Orthodontic Elastics Contaminated In Vitro with Saliva from Healthy Donors: A Pilot Study. Appl. Sci..

[34] Park E.J., Kwon E.Y., Kim H.J., Lee J.Y., Choi J., Joo J.Y. (2018). Clinical and microbiological effects of the supplementary use of an erythritol powder air-polishing device in non-surgical periodontal therapy: A randomized clinical trial. J. Periodontal. Implant Sci..

[35] Fleischer H.C., Mellonig J.T., Brayer W.K., Gray J.L., Barnett J.D. (1989). Scaling and root planing efficacy in multirooted teeth. J. Periodontol..

[36] Fischer C., Wennberg A., Fischer R.G., Attström R. (1991). Clinical evaluation of pulp and dentine sensitivity after supragingival and subgingival scaling. Endod. Dent. Traumatol..

[37] Greenstein G. (1992). Periodontal response to mechanical non-surgical therapy: A review. J. Periodontol..

[38] Boyd L.D., Mallonee L.F., Wyche C.J., Halaris J.F. (2016). Wilkins’ Clinical Practice of the Dental Hygienist.

[39] Montevecchi M., Checchi V., Gatto M.R., Klein S., Checchi L. (2012). The use of a disclosing agent during resective periodontal surgery for improved removal of biofilm. Open Dent. J..

[40] Nepale M.B., Varma S., Suragimath G., Abbayya K., Zope S., Kale V. (2014). A prospective case-control study to assess and compare the role of disclosing agent in improving the patient compliance in plaque control. J. Oral Res. Rev..

[41] Barrows J.N., Lipman A.L., Bailey C.J. (2003). Color additives: FDA’s regulatory process and historical perspectives. Food Saf. Mag..

[42] Allam K.V., Kumar G.P. (2011). Colorants-the cosmetics for the pharmaceutical dosage forms. Int. J. Pharm. Pharm. Sci..

[43] Datta D., Kumar S.R., Narayanan A., Selvamary A.L., Sujatha A. (2017). Disclosing solutions used in dentistry. World J. Pharm. Res..

[44] Block P.L., Lobene R.R., Derdivanis J.P. (1972). A two-tone dye test for dental plaque. J. Periodontol..

[45] Checchi L., Forteleoni G., Pelliccioni G.A., Loriga G. (1997). Plaque removal with variable instrumentation. J.Clin. Periodontol..

[46] Tan A.E., Wade A.B. (1980). The role of visual feedback by a disclosing agent in plaque control. J. Clin. Periodontol..

[47] Petersilka G.J. (2011). Subgingival air-polishing in the treatment of periodontal biofilm infections. Periodontology 2000.

[48] Petersilka G.J., Schenck U., Flemmig T.F. (2002). Powder emission rates of four air polishing devices. J. Clin. Periodontol..

[49] Donnet M., Fournier M., Schmidlin P.R., Lussi A. (2021). A Novel Method to Measure the Powder Consumption of Dental Air-Polishing Devices. Appl. Sci..

[50] Prof Û., Nardi G.M. Système d’aéro-POLISSAGE COMBI Touch. www.mectron.froumectronfrance@mectron.fr.

[51] Momber A., Kovacevic R. (1998). Principles of Abrasive Water Jet Machining.

[52] Barnes C.M. (1991). The management of aerosols with airpolishing delivery systems. J. Dent. Hyg..

[53] Barnes C.M. (2010). An In-Depth Look at Air Polishing.

[54] Petersilka G.J., Bell M., Mehl A., Hickel R., Flemmig T.F. (2003). Root defects following air polishing. J. Clin. Periodontol..

[55] Conserva E., Pisciotta A., Bertoni L., Bertani G., Meto A., Colombari B., Blasi E., Bellini P., de Pol A., Consolo U. (2019). Evaluation of biological response of STRO-1/c-Kit enriched human dental pulp stem cells to titanium surfaces treated with two different cleaning systems. Int. J. Mol. Sci..

[56] Meto A., Conserva E., Liccardi F., Colombari B., Consolo U., Blasi E. (2019). Differential efficacy of two dental implant decontamination techniques in reducing microbial biofilm and re-growth onto titanium disks in vitro. Appl. Sci..

[57] Munro I.C., Berndt W.O., Borzelleca J.F., Flamm G., Lynch B.S., Kennepohl E., Bär E.A., Modderman J. (1998). Erythritol: An interpretive summary of biochemical, metabolic, toxicological and clinical data. Food Chem. Toxicol..

[58] Hashino E., Kuboniwa M., Alghamdi S.A., Yamaguchi M., Yamamoto R., Cho H., Amano A. (2013). Erythritol alters microstructure and metabolomic profiles of biofilm composed of Streptococcus gordonii and Porphyromonasgingivalis. Mol. Oral Microbiol..

[59] Caygur A., Albaba M.R., Berberoglu A., Yilmaz H.G. (2017). Efficacy of glycine powder air-polishing combined with scaling and root planing in the treatment of periodontitis and halitosis: A randomized clinical study. J. Int. Med. Res..

[60] Hägi T.T., Hofmänner P., Salvi G.E., Ramseier C.A., Sculean A. (2013). Clinical outcomes following subgingival application of a novel erythritol powder by means of air polishing in supportive periodontal therapy: A randomized, controlled clinical study. Quintessence Int..

[61] Müller N., Moëne R., Cancela J.A., Mombelli A. (2014). Subgingival air-polishing with erythritol during periodontal maintenance: Randomized clinical trial of twelve months. J. Clin. Periodontol..

[62] Reinhardt B., Klocke A., Neering S.H., Selbach S., Peters U., Flemmig T.F., Beikler T. (2019). Microbiological dynamics of red complex bacteria following full-mouth air polishing in periodontally healthy subjects-a randomized clinical pilot study. Clin. Oral Investig..

[63] Jentsch H.F.R., Flechsig C., Kette B., Eick S. (2020). Adjunctive air-polishing with erythritol in nonsurgical periodontal therapy: A randomized clinical trial. BMC Oral Health.

[64] Hägi T.T., Hofmänner P., Eick S., Donnet M., Salvi G.E., Sculean A., Ramseier C.A. (2015). The effects of erythritol air-polishing powder on microbiologic and clinical outcomes during supportive periodontal therapy: Six-month results of a randomized controlled clinical trial. Quintessence Int..

[65] Tsang Y.C., Corbet E.F., Jin L.J. (2018). Subgingival glycine powder air-polishing as an additional approach to nonsurgical periodontal therapy in subjects with untreated chronic periodontitis. J. Periodontal. Res..

[66] Kargas K., Tsalikis L., Sakellari D., Menexes G., Konstantinidis A. (2015). Pilot study on the clinical and microbiological effect of subgingival glycine powder air polishing using a cannula-like jet. Int. J. Dent. Hyg..

[67] Flemmig T.F., Arushanov D., Daubert D., Rothen M., Mueller G., Leroux B.G. (2012). Randomized controlled trial assessing efficacy and safety of glycine powder air polishing in moderate-to-deep periodontal pockets. J. Periodontol..

[68] Wennström J.L., Dahlén G., Ramberg P. (2011). Subgingival debridement of periodontal pockets by air polishing in comparison with ultrasonic instrumentation during maintenance therapy. J. Clin. Periodontol..

[69] Petersilka G., Koch R., Vomhof A., Joda T., Harks I., Arweiler N., Ehmke B. (2021). Retrospective analysis of the long-term effect of subgingival air polishing in supportive periodontal therapy. J. Clin. Periodontol..

[70] Buser D., Janner S.F., Wittneben J.G., Brägger U., Ramseier C.A., Salvi G.E. (2012). 10-year survival and success rates of 511 titanium implants with a sandblasted and acid-etched surface: A retrospective study in 303 partially edentulous patients. Clin. Implant. Dent. Relat. Res..

[71] Lee C.T., Huang Y.W., Zhu L., Weltman R. (2017). Prevalences of peri-implantitis and peri-implant mucositis: Systematic review and meta-analysis. J. Dent..

[72] Aguirre-Zorzano L.A., Estefanía-Fresco R., Telletxea O., Bravo M. (2015). Prevalence of peri-implant inflammatory disease in patients with a history of periodontal disease who receive supportive periodontal therapy. Clin. Oral. Implants Res..

[73] Renvert S., Hirooka H., Polyzois I., Kelekis-Cholakis A., Wang H.L. (2019). Working Group 3. Diagnosis and non-surgical treatment of peri-implant diseases and maintenance care of patients with dental implants–Consensus report of working group 3. Int. Dent. J..

[74] Ramanauskaite A., Tervonen T. (2016). The Efficacy of Supportive Peri-Implant Therapies in Preventing Peri-Implantitis and Implant Loss: A Systematic Review of the Literature. J. Oral Maxillofac. Res..

[75] Schwarz F., Becker K., Bastendorf K.D., Cardaropoli D., Chatfield C., Dunn I., Fletcher P., Einwag J., Louropoulou A., Mombelli A. (2016). Recommendations on the clinical application of air polishing for the management of peri-implant mucositis and peri-implantitis. Quintessence Int..

[76] Solderer A., Pippenger B.E., Donnet M., Wiedemeier D., Ramenzoni L.L., Schmidlin P.R. (2021). Evaluation of air polishing with a sterile powder and mechanical debridement during regenerative surgical periimplantitis treatment: A study in dogs. Clin. Oral Investig..

[77] Menini M., Setti P., Dellepiane E., Zunino P., Pera P., Pesce P. (2019). Comparison of biofilm removal using glycine air polishing versus sodium bicarbonate air polishing or hand instrumentation on full-arch fixed implant rehabilitations: A split-mouth study. Quintessence Int..

[78] De Siena F., Corbella S., Taschieri S., Del Fabbro M., Francetti L. (2015). Adjunctive glycine powder air-polishing for the treatment of peri-implant mucositis: An observational clinical trial. Int. J. Dent. Hyg..

[79] Lupi S.M., Granati M., Butera A., Collesano V., Rodriguez Y., Baena R. (2017). Air-abrasive debridement with glycine powder versus manual debridement and chlorhexidine administration for the maintenance of peri-implant health status: A six-month randomized clinical trial. Int. J. Dent. Hyg..

[80] John G., Sahm N., Becker J., Schwarz F. (2015). Nonsurgical treatment of peri-implantitis using an air-abrasive device or mechanical debridement and local application of chlorhexidine. Twelve-month follow-up of a prospective, randomized, controlled clinical study. Clin. Oral Investig..

[81] Ji Y.J., Tang Z.H., Wang R., Cao J., Cao C.F., Jin L.J. (2014). Effect of glycine powder air-polishing as an adjunct in the treatment of peri-implant mucositis: A pilot clinical trial. Clin. Oral Implants Res..

[82] Al Ghazal L., O’Sullivan J., Claffey N., Polyzois I. (2017). Comparison of two different techniques used for the maintenance of peri-implant soft tissue health: A pilot randomized clinical trial. Acta Odontol. Scand..

[83] Sahm N., Becker J., Santel T., Schwarz F. (2011). Non-surgical treatment of peri-implantitis using an air-abrasive device or mechanical debridement and local application of chlorhexidine: A prospective, randomized, controlled clinical study. J. Clin. Periodontol..

[84] Persson G.R., Roos-Jansåker A.M., Lindahl C., Renvert S. (2011). Microbiologic results after non-surgical erbium-doped:yttrium, aluminum, and garnet laser or air-abrasive treatment of peri-implantitis: A randomized clinical trial. J. Periodontol..

[85] Hentenaar D.F.M., De Waal Y.C.M., Stewart R.E., Van Winkelhoff A.J., Meijer H.J.A., Raghoebar G.M. (2021). Erythritol airpolishing in the non-surgical treatment of peri-implantitis: A randomized controlled trial. Clin Oral Implants Res..

